# Prospective Preliminary *In Vitro* Investigation of a Magnetic Iron Oxide Nanoparticle Conjugated with Ligand CD80 and VEGF Antibody As a Targeted Drug Delivery System for the Induction of Cell Death in Rodent Osteosarcoma Cells

**DOI:** 10.1089/biores.2016.0020

**Published:** 2016-10-01

**Authors:** AnneMarie Kay Kovach, Jen M. Gambino, Vina Nguyen, Zach Nelson, Taylor Szasz, Jun Liao, Lakiesha Williams, Sandra Bulla, Raj Prabhu

**Affiliations:** ^1^ Agricultural and Biological Engineering Department, Mississippi State University, Mississippi State, Mississippi.; ^2^ College of Veterinary Medicine, Mississippi State University, Mississippi State, Mississippi.

**Keywords:** osteosarcoma, targeted drug delivery, nanoparticles, ligand CD80, VEGF

## Abstract

Target drug deliveries using nanotechnology are a novel consideration in the treatment of cancer. We present herein an *in vitro* mouse model for the preliminary investigation of the efficacy of an iron oxide nanoparticle complex conjugated to vascular endothelial growth factor (VEGF) antibody and ligand cluster of differentiation 80 (CD80) for the purpose of eventual translational applications in the treatment of human osteosarcoma (OSA). The 35 nm diameter iron oxide magnetic nanoparticles are functionalized with an n-hydroxysuccinimide biocompatible coating and are conjugated on the surface to proteins VEGF antibody and ligand CD80. Combined, these proteins have the ability to target OSA cells and induce apoptosis. The proposed system was tested on a cancerous rodent osteoblast cell line (ATCCTM^NPO^ CRL-2836) at four different concentrations (0.1, 1.0, 10.0, and 100.0 μg/mL) of ligand CD80 alone, VEGF antibody alone, and a combination thereof (CD80+VEGF). Systems were implemented every 24 h over different sequential treatment timelines: 24, 48, and 72 h, to find the optimal protein concentration required for a reduction in cell proliferation. Results demonstrated that a combination of ligand CD80 and VEGF antibody was consistently most effective at reducing aberrant osteoblastic proliferation for both the 24- and 72-h timelines. At 48 h, however, an increase in cell proliferation was documented for the 0.1 and 1 μg/mL groups. For the 24- and 72-h tests, concentrations of 1.0 μg/mL of CD80+VEGF and 0.1 μg/mL of VEGF antibody were most effective. Concentrations of 10.0 and 100.0 μg/mL of CD80+VEGF reduced cell proliferation, but not as remarkably as the 1.0 μg/mL concentration. In addition, cell proliferation data showed that multiple treatments (72-h test) induced cell death in the osteoblasts better than a single treatment. Future targeted drug delivery system research includes trials in OSA cell lines from greater phylum species having spontaneous OSA, such as the dog, and on a human OSA cell line model.

## Introduction

Osteosarcoma (OSA) is the most common bone cancer.^1–5^ Patients with OSA have a low survival rate (15–30%) because of distant metastasis, often present at the time of diagnosis.^1,2,4,6^ In addition, patients having undergone tumor excision without supplementary chemotherapy or radiation therapy have an 80% chance of developing metastatic disease.^2^ Possible treatments for OSA consist of surgery, chemotherapy, and radiation therapy.^1–4^ Effective, nonsurgical, minimally invasive therapeutic options for OSA are currently not available, posing a great clinical challenge.

Amputation or limb salvage surgery (LSS) is primary surgical treatments.^1,7^ Survival with amputation is high, but results in low quality of life (QOL).^8^ LSS is considered a better alternative.^8^ Today, 95% of patients undergo LSS rather than amputation.^2^ However, the technique has a higher likelihood of major complications and patients may eventually require amputation regardless.^7–9^

Chemotherapy and radiation therapy are both conventional, modern, and nonsurgical treatment options.^1^ Radiation is not often used for treatment except in inoperable cases.^1,3^ Effects of radiation include leukopenia (low white blood cell counts), which may already be a confounding factor in OSA patients, burns, uneven bone growth, and possible organ dysfunction depending on the area treated.^1^ Caveats to OSA chemotherapy are many. High doses are required to achieve remission, which increases the probability of developing further complications, such as leukopenia that can lead to increased risks of infection and sepsis.^1^ Chemotherapy is typically part of the pre- and postoperative treatment protocol for OSA unless the pathology is minor.^1–4,6^ Frequently, patients do not respond to well-established chemotherapeutic interventions or other treatment protocols, as treatment response can vary depending on the individual.^1,3^ This forces doctors to select a different drug and prescribe the patient another round of chemotherapy, further decreasing his/her QOL. Sustained QOL for OSA patients is a common clinical dilemma regardless of the treatment protocol.

All current treatments for OSA are imperfect and pose risks of harsh side effects and subsequent recrudescence of primary or metastatic cancer.^10–12^ One alternative is targeted drug delivery through nanoparticles.^12–17^ Lack of specificity and negative side effects of conventional chemotherapeutic agents suggests the need for targeting agents. When partnered with a specific targeting agent, drug delivery through nanoparticles has potential to optimize treatment protocols.

Magnetic nanoparticles, such as those used as contrast agents in magnetic resonance imagining, can be manipulated by an external magnetic field.^12–16^ This makes them an optimal choice for targeted drug delivery treatments. Successes using magnetic fields to deliver modified nanoparticles to the tumor sites are described, with the benefit of decreasing necessary chemotherapeutic doses.^15,16^ Studies detailing the modification and engineering of magnetic nanoparticles for targeted drug delivery exist.^12–14^ Using these well-described techniques, the authors of the current report have modified magnetic nanoparticles by way of conjugation to vascular endothelial growth factor (VEGF) antibody and ligand cluster of differentiation 80 (CD80).^13^

Cytotoxic T lymphocyte-associated antigen-4 (CTLA-4), a surface cell receptor that has a natural ligand CD80, is expressed in human OSA tumors.^18,19^ When ligand CD80 comes into contact with a CTLA-4 receptor, an immune response induces apoptosis within OSA cells. Apoptosis occurs through the sequential activation of caspase-8 and caspase-3, which are effectors of the death-receptor-mediated apoptotic signaling pathway.^18,20^ Thus, previous research has established fundamental principles for inducing OSA cell apoptosis.

Herein, we employ the VEGF antibody as the targeting agent because historical studies have demonstrated the presence of VEGF antigen expression in the majority of OSA cell lines examined.^20–22^ The VEGF antigen is responsible for vasculogenesis and is implicated, in general, in tumor growth and metastasis.^21–24^ VEGF increases blood vessel permeability, which leads to blood-borne protein transportation and promotes tumor angiogenesis; both of which lead to further tumor growth and spread.^21–24^ VEGF is expressed in 63.3% of primary OSA tumors and in 70% of metastatic OSA lesions.^20^ Immunohistochemical staining has demonstrated that VEGF pathway genes are amplified in OSA.^25^ Strong evidence for CTLA-4 receptor and VEGF antigen expression in OSA cells has motivated the authors of the current report to incorporate ligand CD80 and VEGF antibody into the proposed system.

The cardinal aim of the research herein was to develop a preliminary *in vitro* approach in an effort to optimize a targeted drug delivery system for OSA treatment. Toward this end, OSA cell line experiments were conducted to ascertain the optimal concentrations of ligand CD80 and VEGF antibody needed on the surface of an iron oxide nanoparticle to reduce cell proliferation using similar technology and methodology as described in previous reports. We describe OSA cell line experiments, gel electrophoresis for verification of conjugation protocol, and the associated efficacy of the proposed targeted drug delivery system. To the authors’ knowledge, no previous studies exist using this technology for the proposed work in the treatment of OSA. We hypothesize as follows: (1) a targeted drug delivery, iron oxide magnetic nanoparticle system, conjugated with ligand CD80 and VEGF antibody and implemented as multiple doses, would significantly reduce *in vitro* rodent OSA cell proliferation and (2) the highest concentration of the two protein conjugates on the nanoparticle surface would be optimal for inducing cell death in this model.

## Materials and Methods

**FIG. 1.**
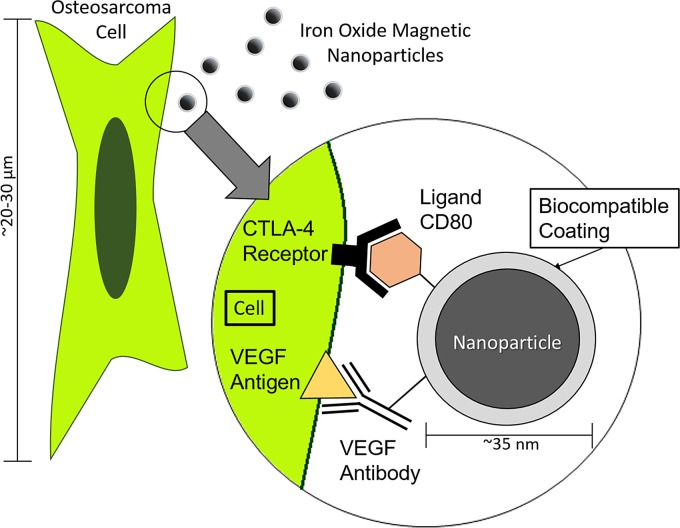
Schematic of a magnetic iron oxide nanoparticle-targeted drug delivery system attached to the surface of an OSA cell by targeted interaction of the VEGF antibody with the VEGF antigen. The interaction of ligand CD80 with the CTLA-4 receptor induces apoptosis in the OSA cell. CD80, cluster of differentiation 80; CTLA-4, cytotoxic T lymphocyte-associated antigen-4; OSA, osteosarcoma; VEGF, vascular endothelial growth factor.

Magnetic iron oxide nanoparticles (OceanNanotech™, San Diego, CA) were used in the creation of a drug delivery system. The nanoparticles arrived prefunctionalized with an n-hydroxysuccinimide (NHS) biocompatible coating. It was then conjugated with VEGF antibody (Sigma-Aldrich, St. Louis, MO) and ligand CD80 (Sino Biological, Inc., Beijing, China) (Fig. 1).

### Nanoparticle conjugation

A protein/ligand solution was made as follows: VEGF antibody was dissolved with the OceanNanotech coupling buffer (ONCB) to concentrations of 0.1, 1.0, 10.0, and 100.0 μg/mL. In a separate solution, ligand CD80 was also dissolved with ONCB to concentrations of 0.1, 1, 10, and 100 μg/mL. A combination of VEGF antibody and ligand CD80 was dissolved with the ONCB to concentrations of 0.1, 1.0, 10.0, and 100.0 μg/mL. A 1-ethyl-3-[3-dimethylaminopropyl]carbodiimide hydrochloride (EDAC) and NHS mixture was made by adding 1 mL of OceanNanotech activation buffer to a preweighed EDAC/NHS mixture tube and mixed by hand to dissolve the solids. A final concentration of 2 mg/mL EDAC and 1 mg/mL NHS was yielded.

Magnetic iron oxide nanoparticles (0.2 mL) were added to a 1.5 mL centrifuge tube, along with 0.1 mL of activation buffer. The EDAC–NHS mixture (100 μL) was added to the nanoparticle solution and mixed using a pipette, yielding a final concentration of 0.5 mg/mL EDAC and 0.25 mg/mL NHS, considered ideal for conjugation by OceanNanotech. The mixture was allowed to react at room temperature (23°C) for 5–10 min with continuous mixing using a digital vortex mixer (VWR, Radnor, PA). ONCB (0.4 mL) was added to the nanoparticle mixture and mixed well. Immediately afterward, protein solution was added to the activated nanoparticle mixture to a maximum volume of 0.5 mL and mixed again. The mixture was allowed to react at room temperature for 2 h with continuous mixing using the digital vortex mixer. OceanNanotech quenching buffer (10 μL) was added, mixed well, and allowed to incubate for 10 min at room temperature.

The reaction mixture was transferred to three 1.5 mL centrifuge tubes. OceanNanotech wash/storage buffer solution (3 mL) was equally divided among the three tubes, and a pipette was used to mix gently. The centrifuge tubes were inserted into the OceanNanotech SuperMag Separator™, and the conjugated nanoparticles were allowed to separate at 4°C for 24 h. All liquid from the tubes was carefully aspirated. Tubes were removed from the magnetic separator. Wash/storage buffer solution (3 mL) was divided between the three centrifuge tubes and the nanoparticles were gently resuspended using a pipette.

The previous steps using the SuperMag Separator to separate the nanoparticles were repeated an additional time, and the conjugated nanoparticles were resuspended with wash/storage buffer solution (1 mL) divided between the three centrifuge tubes and stored for 5–10 days at 4°C.

### Gel electrophoresis

Gel electrophoresis was used to determine conjugation protocol efficacy. Nanoparticles were placed in wells opposing a positive electrode. Five wells were prepared, two with the control (bare nanoparticles) and three containing nanoparticles conjugated with ligand CD80, VEGF antibody, or VEGF+CD80. The gel ran for 30 min at 100 V, and each of the distances was recorded.

### Experimental protocol

Six-well plates were seeded with cells from the rodent OSA cell line (ATCCTM^NPO^ CRL-2836). Seven, six-well plates were seeded, three for each concentration (0.1, 1.0, 10.0, and 100.0 μg/mL) of each protein (ligand CD80, VEGF antibody, and CD80+VEGF) plus an additional plate for the control, which received no treatment. Cells were allowed to settle for 24 h in incubation (34°C with 5% carbon dioxide). Dulbecco's modified essential medium (10% fetal bovine serum, 1% penicillin–streptomycin) (2 mL) was replaced daily in each well.

Osteoblasts were exposed to corresponding protein-conjugated nanoparticles. Nanoparticles (10 μL) were pipetted into each well and agitated gently to ensure even distribution. Wells were incubated for 24 h. After 24 h of exposure, four samples were taken from the first well in each column of the six-well plates and were counted using an automatic cell counter (Bio-Rad Laboratories, Hercules, CA).

The remainder of the cells were fed with medium (2 mL) and treated with a second 10 μL of nanoparticles. The cells were then incubated for another 24 h. After the second 24-h inoculation (a total of 48 h), the wells were again fed with medium (2 mL), counted, and treated with a final 10 μL of nanoparticles. After this 24-h incubation period (a total of 72 h), a final count of remaining cells was obtained.

### Statistical analysis

*t*-Tests were performed using Excel™ (Microsoft, USA) on all cell counts, relative to the control, to test for significance (*n* = 3). A two-tailed, two-sample equal variance test was used. Significance was assigned to differences of *p* ≤ 0.05. Data in tables are presented as average ± standard deviation.

## Results

**FIG. 2.**
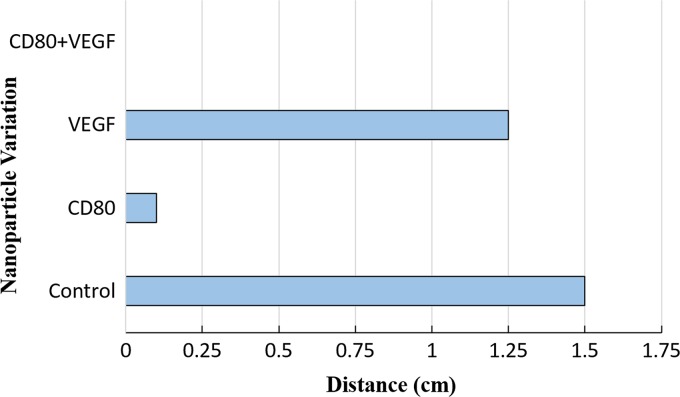
Distance traveled (cm) by multiple nanoparticle systems and control, toward the positive electrode during a gel electrophoresis period of 30 min at 100 V.

Before beginning the OSA cell experiments, gel electrophoresis successfully verified the conjugation protocol (Fig. 2 and Table 1). The nanoparticles conjugated with both ligand CD80 and VEGF antibody (CD80+VEGF) did not travel any distance, if at all, toward the positive electrode. Nanoparticles conjugated with only VEGF antibody traveled 1.25 cm toward the positive electrode. Nanoparticles conjugated with only ligand CD80 traveled 0.10 cm toward the positive electrode. The bare, negatively charged nanoparticles, the control, traveled the furthest (1.50 cm) toward the positive electrode. These findings demonstrate expected results and verify the conjugation protocol used in the experiment.

**Table 1.** **Distance Traveled (cm) by Each Nanoparticle System Toward the Positive Electrode During a Gel Electrophoresis Period of 30 min at 100 V**

Nanoparticle variation	Distance (cm)	Standard deviation
Control	1.5	1.5 ± 0.1
CD80	0.1	0.10 ± 0.1
VEGF	1.25	1.25 ± 0.1
CD80+VEGF	0	0.0 ± 0.1

CD80, cluster of differentiation 80; VEGF, vascular endothelial growth factor.

Four different concentrations (0.1, 1, 10, and 100 μg/mL) were chosen for this preliminary study to find a starting point in narrowing down the range able to effectively induce cell death. A timeline with three points of inoculation, every 24 h, starting at time zero and with three points of measurement (24, 48, and 72 h) was used to assess whether single- or multiple treatments, of the proposed drug delivery system, would be most effective in reducing OSA cell proliferation. A protein conjugate concentration of 1 μg/mL implemented as multiple nanoparticle treatments over 72 h was the most optimal variation for inducing cell death in the rodent OSA cells (Table 2).

**Table 2.** **Live Cell Counts for Each Nanoparticle Exposed Group at 24 h (One NP Treatment), at 48 h (Two NP Treatments), and at 72 h (Three NP Treatments)**

		Average live cell counts (1E+6)		
Nanoparticle variation	Concentration (μg/mL)	24 h	48 h	72 h
Control	—	2.34 ± 0.17	—	5.16 ± 0.26
Ligand CD80	0.10	1.87 ± 0.61	3.74 ± 0.96	0.87 ± 0.16
	1.00	1.54 ± 0.12	1.48 ± 0.47	1.25 ± 0.10
	10.00	2.53 ± 0.51	3.12 ± 2.22	0.78 ± 0.05
	100.00	3.46 ± 0.74	8.47 ± 0.22	0.86 ± 0.17
VEGF antibody	0.10	0.75 ± 0.12	2.85 ± 0.51	0.56 ± 0.06
	1.00	3.09 ± 0.73	3.21 ± 1.59	1.15 ± 0.06
	10.00	2.05 ± 0.12	2.13 ± 0.80	1.15 ± 0.08
	100.00	2.78 ± 0.63	2.32 ± 1.15	1.04 ± 0.09
CD80+VEGF	0.10	1.56 ± 1.09	2.26 ± 1.18	1.07 ± 0.09
	1.00	0.27 ± 0.11	4.30 ± 0.33	0.69 ± 0.08
	10.00	2.27 ± 0.09	1.97 ± 0.11	0.90 ± 0.17
	100.00	1.33 ± 0.93	3.37 ± 0.14	0.81 ± 0.22

NP, nanoparticle.

**FIG. 3.**
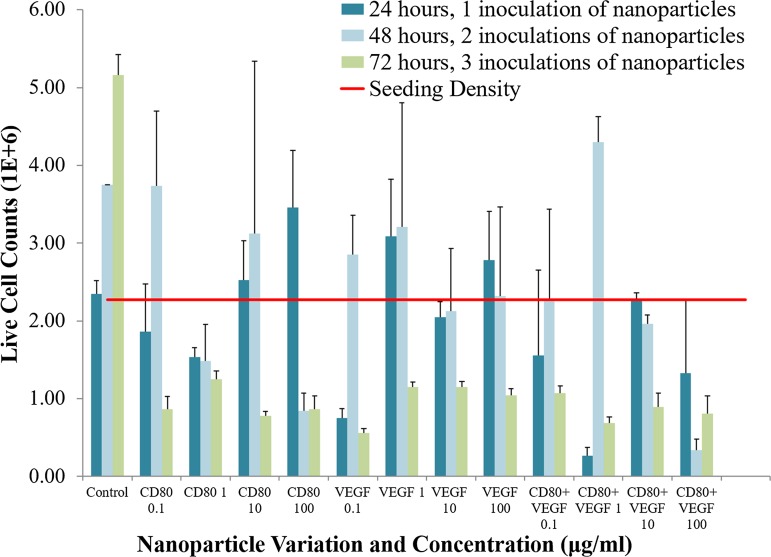
Live cell counts for each NP exposed group at 24 h (one NP treatment), at 48 h (two NP treatments), and at 72 h (three NP treatments) and control. Note that the 48-h control point in all graphs is estimated as the average between 24- and 72-h control data, because of loss of the experimental 48-h control data. Each OSA cell group was exposed to three successive treatments of NP system variations of conjugation to the CD80 and VEGF proteins (ligand CD80, VEGF antibody, and CD80+VEGF) at varying protein concentrations (0.1, 1.0, 10.0, and 100.0 μg/mL). NP, nanoparticle.

After 24 h and one treatment, nanoparticles conjugated with 0.1 μg/mL VEGF antibody and 1 μg/mL CD80+VEGF were most effective in reducing cellular growth (*p* < 0.05), whereas greater concentrations of ligand CD80 (10, 100 μg/mL) and VEGF antibody (1, 100 μg/mL) were least effective (Fig. 3). Furthermore, the least effective protein-conjugated nanoparticle systems did not reduce cell proliferation below the initial seeding density at 24 h (Fig. 3).

For six of the exposed groups, 48-h live cell counts exceeded the initial seeding density. Included in this group were nanoparticle systems that were most effective at 24 and 72 h, such as the 0.1 μg/mL VEGF antibody and 1 μg/mL CD80+VEGF conjugates. Control data were lost for the 48 h because of human error. Despite this, the proliferation beyond seeding density may be an important finding, and for this reason, data from the 48-h counts are presented, yet weighted to a lesser extent.

All nanoparticle exposed groups had cell counts below initial seeding density at 72 h (Fig. 3). However, only three systems had significant differences to the control at 72 h: 1 μg/mL CD80+VEGF, 0.1 μg/mL VEGF antibody, and 10 μg/mL ligand CD80 (*p* < 0.05).

**FIG. 4.**
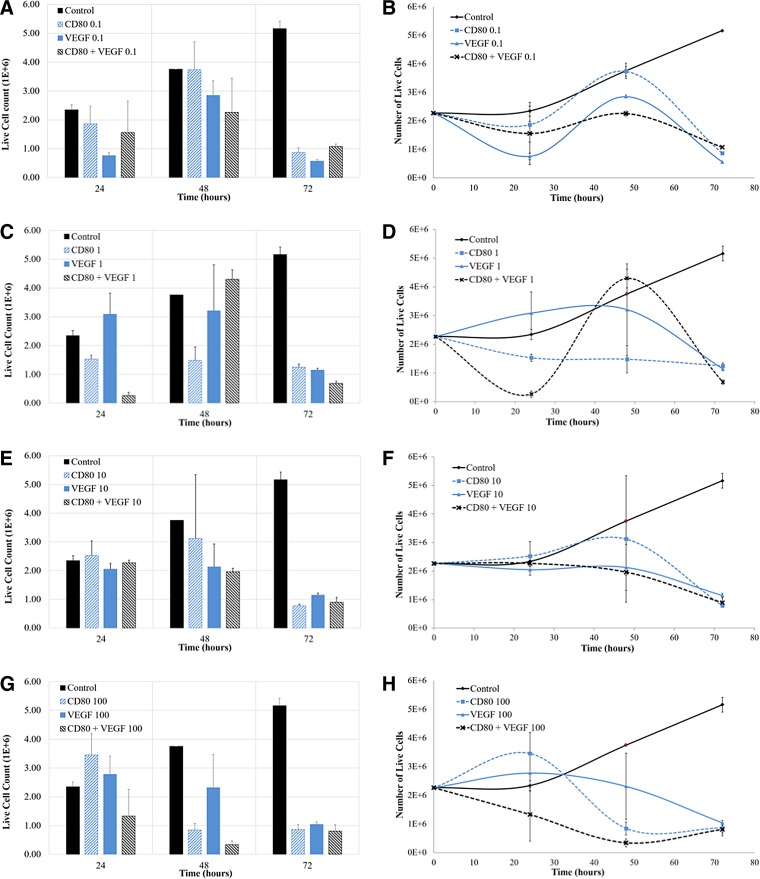
Cell counts and comparisons of NP system efficiency in inducing cell death in rodent OSA cells at 24 h (one NP treatment), 48 h (two NP treatments), and 72 h (three NP treatments). Each pair of bar graphs compares a particular concentration of proteins adhered to the surface of NP systems for the different variations of proteins (ligand CD80, VEGF antibody, and CD80+VEGF). Note that the 48-h control point in all graphs is estimated as the average between 24- and 72-h control data, because of loss of the experimental 48-h control data. This is illustrated as a red point in line graphs.

In transitioning from the 24-h treatment to the 72-h treatment, the only test group that exceeded previous cell counts was CD80+VEGF at 1 μg/mL. At this concentration, cell densities at 24 and 72 h were far below the seeding density (Fig. 3). However, this group also had the highest average cell count at 48 h, denoting a substantial increase in OSA cell proliferation between the second and third nanoparticle treatments. A similar increase was noted in six other test groups (Figs. 3 and 4A–H).

Multiple treatments, measured at 72 h, were more effective than a single treatment after 24 h (*p* < 0.05). Cell proliferation values generally decreased after three treatments of protein-conjugated nanoparticles (Fig. 4A–H).

Cell counts of the different concentrations were compared for each nanoparticle system at 24, 48, and 72 h (Fig. 4A–H). When analyzing the variations in concentrations of protein conjugates, at 24 and 72 h, the 100 μg/mL of each of the CD80, VEGF, and CD80+VEGF was least effective at inducing cell death in OSA cells. A concentration of 1 μg/mL of CD80+VEGF protein-conjugated nanoparticles given in multiple treatments was the optimal system and dosing frequency at arresting OSA cell proliferation. Once the concentration of proteins surpassed 1 μg/mL, a lack of triggered cell death was observed.

## Discussion

This study provides significant data supporting the *in vitro* efficacy of multiple doses of iron oxide nanoparticles conjugated with ligand CD80 and VEGF antibody as a targeted drug delivery system on murine OSA cell line proliferation, supporting our hypothesis. Multiple dosages of different agents are often administered in treatment of OSA patients. The VEGF antibody targets the VEGF antigen on the surface of OSA cells. Ligand CD80 reacts with the CTLA-4 receptors present on the tumor cells to induce apoptosis. We present herein a novel concept of pairing of ligand CD80 with VEGF antibody for *in vitro* use in targeted nanoparticle drug therapy constructs for murine OSA cell lines. The proposed pairing may increase cancer cell selection and lead to greater cell death when used in combination in a specific range of concentrations and with current OSA therapies.

We provide evidence supporting our hypothesis that implementation of multiple, sequential treatments would have greater efficacy given that cell death was greatest when multiple protein-conjugated nanoparticle treatments were delivered than a single exposure. It is plausible that continuing treatments, past what was tested herein, would further decrease or completely arrest rodent OSA proliferation. Future work is needed to test this postulate. Multiple treatments theoretically increase the contact between the drug system and the cancerous cells, making it harder for the cells to sustain growth. Such is typically true for *in vivo* cancer therapeutic protocols as multiple dosages are needed to demonstrate a remission.

Regarding gel electrophoresis, this has not been previously described for the conjugated species developed herein. The control for this test was bare magnetic iron oxide nanoparticles that have a naturally negative charge that becomes more positive when proteins are adhered to the surface. Thus, evaluation of the gel electrophoresis provided verification that the proteins were properly attached to the nanoparticles, lending strength to the study design.

CD80, VEGF, and CD80+VEGF protein-conjugated nanoparticles were all effective at causing cell death in this murine OSA model, aligning with our hypothesis in which we describe the two proteins potentiating OSA kill efficacy. All concentrations of CD80+VEGF demonstrated the greatest efficacy for decreasing the live OSA cell counts, signifying strength of the combination-targeted drug delivery system. Notably CD80+VEGF was most effective at the 1 μg/mL concentration at both 24 and 72 h for the reduction of OSA proliferation. Conjugating the CD80 protein improves efficacy of the nanoparticles, as interaction of this ligand with the CTLA-4 receptor is shown to induce cell death.^18^ Conjugating the VEGF antibody results in increased specificity of the delivery system toward VEGF-positive osteoblasts with CTLA-4, resulting in the best approach for preventing tumor growth and stimulating cell death.

Higher concentrations of VEGF, CD80, and VEGF+ CD80 protein-conjugated nanoparticles were expected to be more effective. For reasons not completely understood, this was not the case in application. Higher concentrations may saturate the nanoparticle system and be counter-productive leading to receptor dysfunction. Phenomena, similar to this, have been described in previously proposed nanoparticle cancer treatment studies.^15,16^ In a study evaluating the efficacy of 1,3-bis(2-chloroethyl)-1-nitrosourea-conjugated nanoparticles for the treatment of intracranial gliomas, a dependency on the concentration of carboxyl acid groups was noted, suggesting that the optimal concentration depends on a number of factors including the maximal loading capacity, biological system, and a balance thereof.^16^ The maximal loading capacity of nanoparticles could be determined by analyzing the surface area to volume ratio and geometric properties of the conjugated nanoparticles.^15,16^ This is a subject of future investigations to optimize the drug delivery system.

Limitations of this work include the loss of control data for the 48-h cell counts and the unexplained increase in treated osteoblast proliferation at that time. This study does not address the potential cytotoxicity of the nanoparticles alone, but such information could provide insight into the proposed system. These issues must be addressed and could have potential ramifications for protein-conjugated nanoparticle OSA treatments in higher phylum species and humans.

Future work will need to address the following issues raised by this study: cell counts were higher at 48 h than at 24 h for seven exposed groups, but subsequently decreased significantly at 72 h. Although 1 μg/mL of CD80+VEGF was very effective, the live cell count of 0.1 μg/mL VEGF at 72 h was marginally lower (*p* = 0.04). This could be because of the confined environment of testing in flasks; *in vivo* testing is needed to determine whether this occurs in living animals. Further studies can investigate titrated doses of the proteins between 0.1, 1, and 10 μg/mL, conjugated to the nanoparticle surface and tested *in vitro* on an OSA cell line. The authors of this report propose additional testing of delivery systems in canine OSA cell lines to answer these questions. Dogs are an ideal translational model given similarities of tumor biology between canine and human OSA. In both, the disease is a spontaneously occurring, primary bone tumor with often-seen complicated clinical ramifications (such as metastasis, pain, and pathological fracture) and bimodal expression.^4^

## Conclusions

In summary, the evidence provided herein suggests that multiple treatments of CD80+VEGF protein-conjugated magnetic iron oxide nanoparticle, drug delivery systems at an optimal concentration of 1 μg/mL, induced the greatest degree of arresting osteoblastic proliferation and inducing cell death for *in vitro* rodent OSA cell lines. We conclude that a single treatment of protein-conjugated nanoparticles would likely be ineffective, but may potentially provide palliation at lower dose intervals. Thus, an ideal concentration between what we established as effective (1 μg/mL) and less effective (0.1, 10, and 100 μg/mL) likely exists and needs to be identified for the sake of greater efficacy.

## Abbreviations Used

CD80: cluster of differentiation 80
CTLA-4: cytotoxic T lymphocyte-associated antigen-4
EDAC: 1-ethyl-3-[3-dimethylaminopropyl]carbodiimide hydrochloride
LSS: limb salvage surgery
NHS: n-hydroxysuccinimide
NP: nanoparticle
ONCB: OceanNanotech™ coupling buffer
OSA: osteosarcoma
QOL: quality of life
VEGF: vascular endothelial growth factor
